# Supplementary material for: Influence of temporary emigration on wood turtle (Glyptemys insculpta) detectability, with implications for abundance estimation
Source: PLoS One. 2024 Apr 16;19(4):e0302170. doi: 10.1371/journal.pone.0302170 (PMC11020976; doi:10.1371/journal.pone.0302170)
Supplement: Attachment — Submitted filename: Beard et al. - PONE-D-23-42768_Response to Reviewers.docx [file pone.0302170.s002.docx]

**Revisions Summary: Influence of temporary emigration on wood turtle (*Glyptemys insculpta*) detectability, with implications for abundance estimation; Allyson N. Beard, Donald J. Brown, Eric T. Hileman, Michael T. Jones, Jena M. Staggs, Ron A. Moen, Andrew F. Badje, Christopher M. Lituma**

We thank the reviewers and associate editor for the time they devoted to their reviews, their close attention to detail, and their suggestions that have resulted in a stronger manuscript. We have addressed the suggestions to the best of our ability. This document is organized by line number in the original submission. For each correction the original sentence is given, followed by the revised sentence. Additional explanations are included when needed. General comments are addressed afterwards.

*(Reviewer 2) the phrase "p_a is random" is used repeatedly in the paper, but its meaning is unclear (and I believe it is used wrongly here). The parameter p_a is a fixed parameter to be estimated, it is not a "random parameter" nor is it even a random effect. I think you mean to say that temporary emigration is random. That's quite different. This needs to be explained somewhere.*

**Original (line 35–36):** The objectives of this study were to determine if *p_a_* is random and if *p_a_* and *p_d_* are influenced by demographic characteristics.

**Revised:** The objectives of this study were to determine if *p_a_* is a random process and if *p_a_* and *p_d_* are influenced by demographic characteristics.

*(Reviewer 2) the statement here that movement out of the survey area causes individuals to be unavailable. In addition to that, individuals being buried in the ground or water also make them unavailable, even if they have not left the survey area.*

*(Reviewer 2) after the current sentence ends at 'individual' you should add 'given that it is available."*

**Original (line 56–62):** Temporary emigration of individuals from a defined survey area is a process that can influence abundance (*N*) estimates (Kendall 1999, Riddle et al. 2010, Wathen et al. 2017). Movement away from the survey area results in an individual being unavailable to be observed during a portion of the sampling events, which is separate from the probability of detecting the individual. For example, portions of a terrestrial salamander population can be subterranean during a sampling event, making them unavailable for detection by surveyors (Bailey et al. 2004).

**Revised:** Temporary emigration of individuals is a process that can influence abundance (*N*) estimates (Kendall 1999, Riddle et al. 2010, Wathen et al. 2017). Temporary emigration can include the movement of individuals outside of survey areas as well as movement within survey areas that results in non-detectability for the sampling design. For example, portions of a terrestrial salamander population can be subterranean during a sampling event, making them unavailable for detection by surveyors (Bailey et al. 2004). Temporary emigration results in an individual being unavailable to be observed during a portion of the sampling events, which is separate from the probability of detecting the individual given that it is available.

*(Reviewer 2) detection given availability is often called perception (I think in some of the Nichols and related work)*

**Original (line 62–64):** Given the presence of an individual in the sampling area at some point during the sampling period, detection (*p*) can be broken down into two components: availability (*p_a_*) and detection given availability (*p_d_*; Riddle et al. 2010).

**Revised:** No changes made.

**Author Response:** While *p_a_* is sometimes referred to as perception, it is most often described as detection given availability. We have chosen to leave it as is to remain consistent with most of the existing literature.

*(Reviewer 2) sentence beginning "These estimates assume..." -- I just don't understand this. Some more detail and clarification is needed.*

**Original (line 67–71):** When separate estimates of *p_d_* and *p_a_* are not possible in populations that experience temporary emigration, *N* refers to the superpopulation size (i.e., the number of individuals that are available for detection at any time during the survey period). These estimates assume that *p_d_* is constant and *p_a_* is random among individuals and surveys, or that model covariates can explain the variation.

**Revised:** When separate estimates of *p_d_* and *p_a_* are not possible in populations that experience temporary emigration, *N* refers to the superpopulation size (i.e., the number of individuals that are available for detection at any time during the survey period), with the implicit assumption that pd is constant and pa is random among individuals and surveys, or that model covariates can explain the variation.

*(Reviewer 1) In the intro it would be necessary to introduce some peculiarities of the natural history of turtles, including their high inter-annual survival, since this is relevant to interpret the results of this study and totally differs from other small ectothermic vertebrates.*

**Original (75–80):** The wood turtle (*Glyptemys insculpta*) is a semi-aquatic freshwater turtle and species of conservation concern endemic to east-central North America (Garber and Burger 1995, Daigle and Jutras 2005, van Dijk and Harding 2011, Willoughby et al. 2013). The species is currently categorized as threatened by the Committee on the Status of Endangered Wildlife in Canada (COSEWIC 2018) and is under review for listing under the U.S. Endangered Species Act (U.S. Fish and Wildlife Service 2016).

**Revised:** The wood turtle (*Glyptemys insculpta*) is a semi-aquatic freshwater turtle and species of conservation concern endemic to east-central North America (Garber and Burger 1995, Daigle and Jutras 2005, van Dijk and Harding 2011, Willoughby et al. 2013). Unlike many small vertebrates, wood turtles have long life spans with high adult annual survivorship (typically >0.9) and delayed sexual maturity (11 – 20 years of age; Willey et al. 2021). This life history strategy is likely not conducive to a world with rapid anthropogenic change (Stanford et al. 2020, Roberts et al. 2021). Wood turtles are currently categorized as threatened by the Committee on the Status of Endangered Wildlife in Canada (COSEWIC 2018) and is under review for listing under the U.S. Endangered Species Act (U.S. Fish and Wildlife Service 2016).

*(Reviewer 2) The N-mixture model context comes up rather abruptly in the Discussion, and the paper is not about the N-mixture model so this is a little confusing. You might introduce the relevant context in the Introduction. Also, FWIW, the stratified N-mixture model is well suited for data collected by size class. One paper about this class of models is here:*

*Zipkin, E.F., Thorson, J.T., See, K., Lynch, H.J., Grant, E.H.C., Kanno, Y., Chandler, R.B., Letcher, B.H. and Royle, J.A., 2014. Modeling structured population dynamics using data from unmarked individuals. Ecology, 95(1), pp.22-29.*

*(Reviewer 2) 'temporary emigration is random or directional' -- I think these concepts need defined (or else I missed that perhaps).*

**Original (line 94–96):** Whether this temporary emigration is random or directional has not been assessed. Therefore, it is unclear if current *N* estimates, which assume random temporary emigration for all individuals, accurately represent the superpopulation size.

**Revised:** Currently, *N*-mixture models are the most common method used to estimate *N* using wood turtle monitoring program data (e.g., Brown et al. 2017, Roberts et al. 2021). Key assumptions of *N*-mixture models include that all individuals have the same detection probability and that the population is closed, or availability is a random process during the sampling period (Kéry and Royle 2016). Whether the temporary emigration of wood turtles during the survey period is a random or Markovian process (i.e., dependent on the previous state) has not been assessed (sensu Kendall et al. 1997). Therefore, it is unclear if current *N* estimates accurately represent the superpopulation size.

**Original (lines 201–203)**: A key assumption of *N*-mixture models, the abundance estimation method typically used for analyses of wood turtle monitoring program data (Brown et al. 2017, Roberts et al. 2021), is that all individuals have the same detection probability.

**Revised**: A key assumption of *N*-mixture models, the abundance estimation method typically used for analyses of wood turtle monitoring program data (Brown et al. 2017, Roberts et al. 2021), is that all individuals have the same detection probability (although we note that customization of *N*-mixture models to incorporate stage-specific parameter estimates is possible; Zipkin et al. 2014).

*(Reviewer 1) In the introduction it would be advisable for the authors to include references that support their hypothesis that pa and pd could be influenced by demographic traits*

*(Reviewer 2) here again is the statement that 'p_a is random' (see comment above)*

*(Editor) I think it would also be helpful to set up some expectation or predictions in the introduction to which the results could be compared. For example, did you expect size to influence these parameters and why?*

**Original (line 97–99):** Our objective was to assess sampling assumptions for existing wood turtle survey protocols (Brown et al. 2017, Northeast Wood Turtle Working Group 2021), specifically to determine if *p_a_* is random and whether *p_a_* and *p_d_* are influenced by demographic characteristics.

**Revised:** Our objective was to assess sampling assumptions for existing wood turtle survey protocols (Brown et al. 2017, Northeast Wood Turtle Working Group 2021), specifically to determine if *p_a_* is a random process and whether *p_a_* and *p_d_* are influenced by demographic characteristics. Previous studies that tracked locations of individual turtles suggest that movement and habitat use patterns differ between males and females (e.g., Kaufmann 1995, Brown et al. 2016, Hagani et al. 2021), and it is assumed that smaller turtles are harder to detect during surveys (Jones et al. 2018). Thus, we hypothesized that *p_a_* would be influenced by turtle sex and *p_d_* would be influenced by turtle size.

*(Reviewer 2) this description of the double pass approach is not clear. Does this mean each transect was run twice? i.e., down and back? This is my understanding, but it is confusing because of "bands" and then repeated "for each transect" within "each primary". It's just not clear. Please work on the wording here and maybe add a hypothetical search line to Figure 2, in a different color.*

*In any case, about this double pass approach, I think this has to produce violations of basic assumptions. If you catch a turtle on the first pass, you are more likely to catch it on the 2nd pass (or perhaps less likely). There is some behavioral response of both the observer and the individual turtle. This has to be discussed and dealt with in some manner.*

*Certainly if the turtle did not move, the 2nd detection would not be stochastic but, rather, would occur with probability 1. (this is like a removal design in a sense).*

**Original (line 126–131)**: We modified the protocol for this study to include one additional pass along each transect band. Immediately after completion of the first pass, the observers switched transects and continued searching the other transect band on the same side of the river. The same process was repeated for the other side of the river. This resulted in a double pass (i.e., a secondary survey period) for each transect within each primary survey period, thus allowing the populations to be considered closed between passes and open between survey days.

**Revised**: We modified the protocol for this study to include one additional pass along each transect band by an independent observer. Immediately after completion of the first pass, the observers switched transect bands and conducted a second survey on the same side of the river. The same process was repeated for the other side of the river. This resulted in a double pass (i.e., a secondary survey period) for each transect band within each primary survey period, thus allowing the populations to be considered closed between passes and open between survey days.

*(Reviewer 2) insert "survival probability" after "fixed" and before "S"*

**Original (line 150–151):** We also fixed S at 1 because we assumed adult survival probability was near 1 during the 5-week survey window.

**Revised:** We also fixed survival probability (*S*) at 1 because we assumed adult survival probability was near 1 during the 5-week survey window.

*(Reviewer 2) I don't understand this gamma prime and gamma double prime notation. What does that mean? Why not use t and t-1 here? Define/explain please.*

**Original (line 159–161):** The Markovian *γ* structure assumed that availability for detection at time *t* was linked to availability at time *t* - 1 (𝜸‵ ≠ 𝜸‶), whereas the random model assumed that availability status at time *t* was not influenced by previous availability status (𝜸‵ = 𝜸‶).

**Revised:** The Markovian *γ* structure assumed that availability for detection at time *t* – 1 influences availability at time *t* (𝜸‵ ≠ 𝜸‶*;* the probability of being outside the search area during the next survey is different for individuals inside the search area compared to those already outside the search area during the current survey), whereas the random model assumed that availability status at time *t* was not influenced by the previous availability status (𝜸‵ = 𝜸‶; every individual in the superpopulation has the same probability of being outside the search area during the next survey).

*(Reviewer 2) 'Markovian \gamma structure' what does this mean?*

**Original (line 167):** We also assessed if there was support for a Markovian *γ* structure for only males, females, or juveniles

**Revised:** We also assessed if there was support for a Markovian temporary emigration structure for only males, females, or juveniles (Table 1).

**Author Response:** Markovian is defined on lines 93 and lines 190 **–** 194 in the revised manuscript.

*(Reviewer 2) "carapace length as a covariate" -- for both parameters? If so be explicit about that.*

**Original (line 174–177):** The most parsimonious *p* and *c* model from the first set of candidate models included carapace length as a covariate (AIC*c* = 910.479; *wi* = 0.622), with age class also receiving strong support (ΔAIC*c* = 1.03; *wi* = 0.371).
**Revised:** The most parsimonious *p* and *c* model from the first set of candidate models included carapace length as a covariate for both parameters (AIC*c* = 910.479; *wi* = 0.622), with age class also receiving strong support (ΔAIC*c* = 1.03; *wi* = 0.371).

*(Reviewer 2) The phrase "Markovian gamma structure" is used here. You might as well just say "Markovian temporary emigration structure" because that is what is meant.*

**Original (line 181–182):** The top 4 models of the second candidate model set included a Markovian *γ* structure (Table 2; ∑*w_i_* = 0.815). Both the sex and age class *γ* models had substantial support.

**Revised:** The top 4 models of the second candidate model set included a Markovian temporary emigration structure (Table 2; ∑*w_i_* = 0.815). Both the sex and age class temporary emigration models had substantial support.

*(Reviewer 2) I think there is redundant notation: p_d and p_a are used to define parameters but also in the text the terms p and c are used to denote capture and recapture. How are these related to p_d and p_a ?*

**Original (line 141–143):** We estimated capture probability (*p*), recapture probability (*c*), and availability for detection (1-*γ*) using a robust design model with the Huggins’ estimator (Table 1; model ‘Robust Design Huggins’ *p* and *c*’; Kendall and Bjorkland 2001).

**Revised:** We estimated capture probability (*p_dc_*; *p* in Program MARK), recapture probability (*p_dr_*; *c* in Program MARK), and availability (*p_a_*;1-*γ* in Program MARK) using a robust design model with the Huggins’ estimator (Table 1; model ‘Robust Design Huggins’ *p* and *c*’; Kendall and Bjorkland 2001).

**Author Response:** Comment originally made on line 188. *p* and *c* changed to *p_dc_* and *p_dr_* throughout the manuscript to follow the *p_d_* notation.

*(Reviewer 1) The authors must explain what the Midwest protocol is, since Plos ONE is an international journal and most readers (included me) not know what this protocol consists of.*

*What has been the p previously reported in the Midwest protocol?*

**Original (line 188–190):** Our results indicate that detection given availability (*p_d_*) is higher than detection probabilities (*p*) that have been previously reported using the Midwest protocol. Brown et al. (2017) reported a maximum detectability of approximately 0.2 during ideal conditions.

**Revised:** Our results indicate that detection given availability (*p_d_*) is higher than detection probabilities (*p*) that have been previously reported using the Midwest protocol. Brown et al. (2017) reported a maximum detectability of approximately 0.2 during ideal conditions using the original protocol.

**Author Response:** The Midwest protocol is described in lines 83–90 and 128–134 in the revised manuscript.

*(Reviewer 1) Authors should explain why they believe LCL alters the probability of detection, citing appropriate references.*

**Original (line 196–198):** Based on our top model from the first candidate model set, carapace length is a strong driver of *p_d_*. In our top model overall, estimates of *p_d_* increase from 0.32 for the smallest recorded turtle size (57 mm) to 0.63 for the largest recorded turtle size (234 mm).

**Revised:** Based on our top model from the first candidate model set, carapace length is a strong driver of *p_d_*. In our top model overall, estimates of *p_d_* increase from 0.32 for the smallest recorded turtle size (57 mm) to 0.63 for the largest recorded turtle size (234 mm), supporting previous qualitative observations (Jones et al. 2018). While there may be habitat use differences between adults and juveniles that influence pd, larger turtles are more conspicuous and thus are more likely to be seen during surveys.

*(Reviewer 2) here the term 'directional movement' is used. This needs defined and related to previous concepts discussed in this paper.*

**Original (line 209–210):** There was stronger evidence for directional movement than random movement throughout the survey period.

**Revised:** There was stronger evidence for Markovian movement than random movement throughout the survey period.

**Author Response:** The term ‘directional movement’ was changed to ‘Markovian movement’ throughout the manuscript to help with clarity and consistency.

**General Comments**

*(Reviewer 2) Data collection: The protocol used here would be ideal to apply spatial capture-recapture models since you have both spatial information about the search and the capture locations.*

**Author Response:** We appreciate the reviewer’s suggestion to consider spatial capture-recapture models. While these models can be very useful for improving our understanding of habitat associations and fine-scale density estimation, they do not fit well within the context of the standardized wood turtle monitoring programs, where the unit of inference is the monitoring site and the objective is to track site-level trends over time. Since the objective of this study is to provide information that can be used to improve either the protocols or the standard data analysis approach, we retained our original analytical choice. However, we do intend to explore the use of SCR models for future habitat selection research.

(*Reviewer 1) In general, both the discussion and the intro are very focused on the target species, Glyptemys insculpta. Plos ONE is a generalist and not a herpetological journal, the authors should work on generalize their discussion and conclusions.*

**Author Response:** The first 3 paragraphs of the Introduction contain general information on the study topic and for the revised manuscript we have chosen to only modify the introduction per the specific reviewer suggestions. We agree with the reviewer that the discussion is very focused on the target species and would benefit from a discussion of broader implications, and have added an additional Discussion paragraph to the paper:

While our study focused specifically on investigating assumptions of a survey protocol for the wood turtle, many other species monitoring programs use count data from spatially constrained VES protocols to estimate occurrence or abundance, such as for eastern box turtles (*Terrapene carolina*) in Massachusetts (Erb et al. 2015), foothill yellow-legged frogs (*Rana boylii*) in California (Peek et al. 2017), and Shenandoah salamanders (*Plethodon shenandoah*) in Virginia (Grant et al. 2021). Every species monitoring program operates with limited resources and the associated survey protocols reflect tradeoffs between intensity of sampling at individual sites and amount of spatial coverage across the monitoring area. While there are many different statistical frameworks and models available for VES data analyses, all models have assumptions and it is important to consider, and if needed, experimentally test whether the data being collected are appropriate for the anticipated statistical analysis. While it will not always be possible to modify protocols to meet every model assumption, in some cases minor modifications to the sampling protocol or statistical analyses could improve accuracy of the resulting model estimates, thus improving quality of the information used to guide species management decisions and actions.
